# Clinical behavior of recurrent pleomorphic adenoma in the palate: a systematic review

**DOI:** 10.1590/acb390824

**Published:** 2024-02-26

**Authors:** Gabriela Lopes-Santos, Najara Gomes de Oliveira Marques, Kellen Cristine Tjioe, Denise Tostes Oliveira

**Affiliations:** 1 Universidade de São Paulo – Faculdade de Odontologia de Bauru – Departamento de Cirurgia, Estomatologia, Patologia e Radiologia – Bauru (SP) – Brazil.; 2 Augusta University – Georgia Cancer Center – Augusta (GA) – United States of America.

**Keywords:** Neoplasms, Adenoma, Pleomorphic, Salivary Glands, Recurrence

## Abstract

**Purpose:**

This systematic review analyzed the clinical behavior and odds of malignancy of the palatal recurrent pleomorphic adenomas.

**Methods:**

Systematic review of patients with recurrent pleomorphic adenoma arising in the palate. Database search: MEDLINE, Scopus, Web of Science, Cochrane, EMBASE, Virtual Health Library, Google Scholar, and OpenGrey. A binomial logistic regression was performed to assess the odds of detecting recurrence five, 10 and 20 years after the treatment of primary tumor.

**Results:**

Thirteen studies (n = 18 patients) out of 336 were included. The recurrent pleomorphic adenoma in palate was more common in females (61.6%), average age was 49 years old (range 9–73 years old). Four patients progressed to malignant transformation. The *odds ratio* (OR) of detecting a recurrence at 10 (OR = 5.57; 95% confidence interval – 95%CI 1.13–27.52), and 20 years (OR = 18.78; 95%CI 3.18–110.84) after treatment of primary pleomorphic adenoma was significantly higher than at one-year follow-up.

**Conclusions:**

The recurrence of pleomorphic adenoma in palate remains a rare event of late occurrence. It mainly affects middle-aged female and carries a risk of malignant transformation. Although uncommon, patients with palatal pleomorphic adenoma should be warned about the possibility of recurrence or malignant transformation of tumor at advanced ages.

## Introduction

Pleomorphic adenoma is the most common type of benign salivary gland tumor and may develop in the major and in the minor salivary glands^1^^,^ 2. Approximately more than 30% of the pleomorphic adenomas arise in the minor salivary glands, and half of these cases occur in the palate^2^^,^ 3. Clinically, pleomorphic adenoma in palate has good prognosis, and it typically appears as a slow growing and asymptomatic solitary nodule, completely or partially surrounded by capsule, that may cause compressive bone resorption^1^. Despite of most palatal pleomorphic adenomas are partially or nonencapsulated^3^^-^ 6, its recurrence after surgical treatment varies from 2% at five years to 7% at 20 years of follow-up time^7^.

Efforts to understand the factors involved in the recurrence of pleomorphic adenoma have been the focus of diverse studies and systematic reviews^8^^-^ 10. Some features such as gender, age of the patient at diagnosis, duration of the tumor, incomplete surgical resection, deficient encapsulation, and previous radiotherapy have been suggested as risk factors for recurrent pleomorphic adenoma (RPA)^4^^-^ 6. However, due to the limited number of reports of RPA in minor salivary glands, the studies often include tumors affecting major and minor salivary glands^9^^,^ 11.

Similarly, the malignant transformation of oral pleomorphic adenoma occurs in less than 5% of cases and is associated to multiple recurrences and longer time of development of the lesion^12^^-^ 14. Nevertheless, most factors associated with recurrence and/or malignancy of pleomorphic adenoma of the mouth is based in studies involving mainly the parotid gland, the most frequently affected site by this tumor^1^^,^ 9 ^,^ 14 ^-^ 16. Studies evaluating the clinical behavior of RPA arising in minor salivary glands are scarce^9^^,^ 17 ^,^ 18.

Although the pleomorphic adenoma in palate has a good prognosis, determinants regarding its clinical behavior when recurrence or malignant transformation occur are unclear^7^^,^ 14 ^,^ 15. Thus, the aim of this systematic review was to analyze the case reports of RPA to detect potential factors that influence the recurrence, clinical behavior, and the odds of malignancy of this tumor in palate.

## Methods

The scientific question addressed by this study was: what are the factors that influence the clinical behavior of palatal pleomorphic adenoma that relapsed or underwent malignant transformation after the treatment? This systematic review was conducted in accordance with the Preferred Reporting Items for Systematic Review and Meta-Analyses (PRISMA) 2020 statement guidelines^19^. The protocol was registered in International Prospective Register of Systematic Review (PROSPERO) database under the number CRD42023398419.

### Eligibility criteria

The inclusion criteria followed the Patient, Intervention, Comparison, Outcome, and Study design (PICOS) strategy, as follows:

- Population: patients with recurrent pleomorphic adenoma arising in the palate;
- Intervention: clinicopathological features of the patients, treatment and time of tumor recurrence;
- Comparison: none;
- Outcome measures: tumor recurrence after treatment of primary tumor in the palate (with or without malignant transformation);
- Types of study: cross-sectional studies, case series, case reports, letters to the editor, short communications.

The exclusion criteria were:

- Studies reporting RPA in anatomic sites other than palate or without description of histopathological confirmation;
- pre-clinical and *in-vitro* studies;
- Clinical studies or cases series of RPA lacking key information about clinicopathological features individualized of the patients, treatment, or clinical outcome of patient.

### Information sources and search strategy

The literature search was carried out in the following electronic databases: PubMed/MEDLINE, Scopus, Web of Science, Cochrane Library, and EMBASE. In addition, the grey literature was also queried manually using the databases Virtual Health Library, OpenGrey, and Google Scholar (the 100 first results). The search strategy was adapted for each database (Suppl. Mat. 1). All searches were performed on March 20^th^, 2023. There were no limitations on the language and year of publication of the articles.

### Study selection

All studies retrieved were imported into the reference management tool EndNote Web (EndNote Web; Thomson Reuters Inc., Philadelphia, PA, United States of America), and the duplicates were excluded. The authors conducted a thorough review of previous findings to confirm adherence to the inclusion criteria. In the first phase of the study selection, two independent reviewers analyzed the titles and abstracts of the studies to screen the relevant articles. Any disagreements were resolved by discussion, and an expert in oral pathology was consulted when necessary. In the second phase, the same reviewers read the full texts of the studies selected in the phase 1 regardless applying the inclusion and exclusion criteria. If there were any disagreements, the expert was consulted.

### Data extraction

Two authors extracted the relevant information of the studies into a spreadsheet. Data collected included: study design, country, number of patients, demographic characteristics of patients (sex and age), clinicopathological features of the tumor (clinical description, size, anatomic location, imaging exams, and treatment), and clinical outcome (time of recurrence after the initial treatment and/or malignant transformation). All data was consistently coded across all studies.

### Risk of bias assessment

The Joanna Briggs Institute (JBI) Critical Appraisal Checklist for case reports was utilized to evaluate the risk of bias of the individual studies^20^. The JBI checklist included questions regarding the patient demographics, clinical history and presentation, diagnostic assessments, treatments, post-treatment condition, adverse events, and key takeaway points. The assessments were conducted independently by two reviewers. Any disagreements were resolved by consensus, and a third reviewer was consulted when necessary.

### Synthesis methods

All statistical analyses were performed using the Statistical Package for Social Sciences, Version 26.0 (Chicago, IL, United States of America), and GraphPad Prism version 8.0 (GraphPad Software, San Diego, CA, United States of America).

The clinical features of patients with RPA in palate were reported in frequency of cases, percentages, mean of ages, mean of tumor recurrence time, and mean of malignancy time. A binomial logistic regression was performed to assess the *odds ratio* (OR) of detecting recurrence at one, five, 10, and 20 years after treatment of primary pleomorphic adenoma (categorical variables). The confidence interval (CI) was set at 95%. The association between the mean tumor recurrence time and malignancy was verified by Fisher’s exact test. Values of p < 0.05 were considered significant for all statistic tests.

## Results

### Study selection

**Figure 1:**
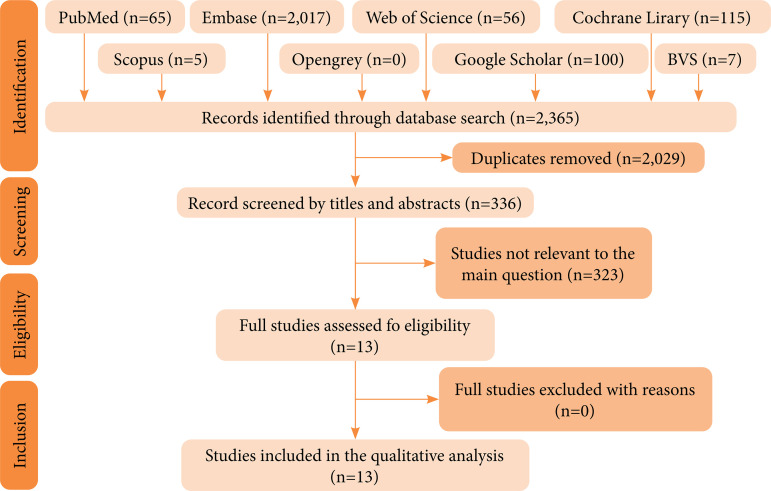
Flowchart screening the steps for the articles included in this systematic review.

The electronic search returned the total of 2,365 articles. After duplicates removal, 336 manuscripts were screened based on their titles and abstracts. Thirteen articles met the eligibility criteria and were selected for full-text analysis. All 13 articles fulfilled the inclusion criteria and were included in this review: two retrospective studies^21^^,^ 22, 10 case reports^23^^-^ 32, and one letter to the editor^33^. All 13 studies were published in English, between 1957 and 2022, and were conducted in United Kingdom^24^^,^ 25 ^,^ 33, India^29^^,^ 32, United States^22^^,^ 23, Japan^21^, Greece^26^, Iran^27^, Italy^28^, South Africa^31^, and Brazil^30^. The flowchart shows the selection process (Fig. 1).

BVS: Virtual Health Library. Source: elaborated by the authors.

### Characteristics of the included studies

The characteristics of the 13 included studies are summarized in Table 1. The sample included 18 patients with RPA in palate. Regarding gender, 11 (61.1%) patients were females and seven (38.9%) males. The mean age of patients with RPA was 49.3 years old (range 9–73 years old). In patients who developed RPA, the mean age of the primary tumor was 33 years old (range 4–68).

**Table 1:** Summary of the main clinicopathological characteristics of the patients with recurrent pleomorphic adenoma included in this systematic literature review.

Author, year	Gender	Primary tumor (age of patient)	Recurrent tumor (age of patient)	Time for relapse	Clinicalfeatures	Bone involvement	Treatment	Finaldiagnosis
Byars et al., 1957^23^	Female	4 years old	9 years old	Five years	NA	NA	Surgical resection	Recurrent pleomorphic adenoma
Hellquist and Michaels, 1986^24^	Female	25 years old	55 years old	30 years	Protruding and partly ulcerated with small areas of hemorrhage, measuring 20 × 15 × 15 mm	Yes	Radiotherapy and palatal resection	Chondrosarcoma
	Male	28 years old	64 years old	36 years	Mass firm and cyst-like measuring 13 × 7 × 5 mm	NA	Radiotherapy and palatal resection	Carcinoma of ductal type
Yasumoto et al., 1999^21^	Male	48 years old	57 years old	Nine years	Solitary, smooth measuring approximately 40 mm	Yes	Surgical resection	Recurrent pleomorphic adenoma
	Female	46 years old	58 years old	12 years	Solitary, smooth measuring 42 mm	Yes	Surgical resection	Recurrent pleomorphic adenoma
	Female	24 years old	64 years old	40 years	Solitary, smooth measuring approximately 40 mm	Yes	Surgical resection	Recurrent pleomorphic adenoma
	Male	43 years old	66 years old	23 years	Solitary, smooth measuring 66 mm	Yes	Surgical resection	Recurrent pleomorphic adenoma
	Male	62 years old	69 years old	Seven years	Solitary, smooth measuring 66 mm	No	Surgical resection	Recurrent pleomorphic adenoma
Shaaban et al., 2001^25^	Male	7 years old	9 years old	Two years	Non-tender, immobile and showed an ulcerated surface measuring 2 × 2 cm	No	Surgical resection	Recurrent pleomorphic adenoma
Karatzanis et al., 2005^26^	Female	68 years old	70 years old	Two years	Large tumor, arising from the right side of the soft palate, and blocking completely the oral cavity measuring 4.6 × 6 cm	NA	Surgical resection	Low-grade malignant myoepithelial carcinoma
Turner and Smith, 2006^33^	Female	19 years old	54 years old	35 years	Solitary, smooth measuring 1.2 cm	Yes	Partial maxillectomy	Recurrent pleomorphic adenoma
Negahban et al., 2006^27^	Male	23 years old	53 years old	30 years	Slowly growing mass with rapid growth measuring 2.5 × 1.5 × 1 cm	NA	NA	Clear cell carcinoma
Berardi et al., 2009^28^	Male	20 years old	39 years old	19 years	Swelling measuring 1.2 cm	NA	Surgical resection	Recurrent pleomorphic adenoma
Ritwik and Brannon, 2012^22^	Female	14 years old	17 years old	Three years	Swelling measuring 1.3 cm	Yes	Surgical resection	Recurrent pleomorphic adenoma
Faisal and Mishra, 2016^29^	Female	53 years old	73 years old	20 years	Facial deformity with expansion of bilateral maxillae and palatine bones, deformed nasal bones	Yes	Surgical resection	Recurrent pleomorphic adenoma
Lima et al., 2020^30^	Female	36 years old	46 years old	10 years	Nodular firm measuring 2.2 × 3 cm	No	Surgical resection	Recurrent pleomorphic adenoma
Hemavathy et al., 2022^32^	Female	50 years old	58 years old	7.5 years	Smooth, firm, non-tender, fixed swelling over the anterior maxilla with 3 × 2 cm of diameter	NA	Surgical resection	Recurrent pleomorphic adenoma
Nokaneng, 2022^31^	Female	24 years old	28 years old	Four years	Asymptomatic, fluctuant swelling with 3 × 2 cm of diameter	Yes	Surgical resection	Recurrent pleomorphic adenoma

NA: data not available. Source: Elaborated by the authors.

RPA in the palate was clinically characterized as a painless, solitary well-defined and firm nodule, usually measuring up to 3 cm in diameter^21^^,^ 22 ^,^ 28 ^-^ 33. The tumor was often covered by normal-colored oral mucosa with occasional superficial ulceration^25^. The presence of bone involvement was reported in nine cases^21^^,^ 22 ^,^ 24 ^,^ 29 ^,^ 31 ^,^ 33. In most of patients, the magnetic resonance imaging or cone-beam computed tomography revealed a hypodense maxillary lesion, sometimes with irregular margins and extending to the maxillary sinus, nasal, and orbital cavity^21^^,^ 22 ^,^ 24 ^,^ 29 ^,^ 31 ^-^ 33.

### Clinical outcome

The tumor recurrence time ranged from two to 41 years (mean 16.3 years) after treatment of the primary pleomorphic adenoma in the palate. The surgical removal was the treatment of choice in all cases^21^^,^ 22 ^,^ 24 ^-^ 33. Two cases, that were managed with radiotherapy following the surgery of the primary tumor, underwent malignant transformation^24^.

Four out of the 18 patients with RPA in the palate underwent malignant transformation^24^^,^ 26 ^,^ 27. Therefore, the malignant transformation rate among patients with RPA was 22.2%, and the time of tumor progression before the malignancy diagnosis ranged from two to 36 years (mean of 23 years). The malignant transformation of RPA in palate occurred 30 years after the primary tumor removal, in three out of the four patients. Among the four patients with RPA who developed malignant tumors, only one developed a tumor of mesenchymal origin, specifically chondrosarcoma^24^, whereas the remaining three patients developed tumors of epithelial origin, including one clear cell carcinoma^27^, one carcinoma of ductal type^24^, and one low-grade malignant myoepithelial carcinoma^26^. All patients with malignant tumors were treated with surgery^24^^,^ 26, and adjuvant radiotherapy was performed in two patients^24^ (Table 1).

### Risk of bias in studies

The results of the risk of bias of cases reports of RPA included are available as supplementary data (see Data Availability Statement section). Six studies demonstrated low risk of bias^25^^,^ 26 ^,^ 28 ^,^ 30 ^-^ 32, six studies demonstrated unclear risk of bias^21^^,^ 23 ^,^ 24 ^,^ 27 ^,^ 29 ^,^ 33, and one study demonstrated high risk of bias^22^.

### Results of quantitative synthesis

**Figure 2:**
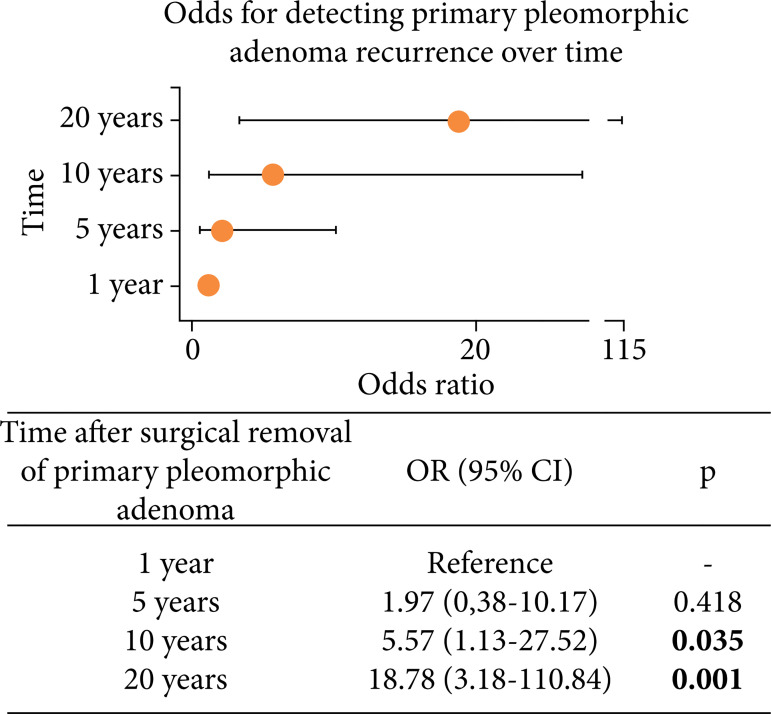
The odds for detecting primary pleomorphic adenoma recurrence over time. Bold values denote statistical significance.

Source: Elaborated by the authors.

The OR of detecting a recurrence 10 and 20 years after the treatment of the primary pleomorphic adenoma was 5.57 (95%CI 1.13–27.52) and 18.78 (95%CI 3.18–110.84) times higher than at the one-year follow-up time, respectively (Fig. 2).

No statistically significant association was detected between mean time of tumor recurrence (< 16 years or ≥ 16 years) and malignant transformation (p = 0.5865). However, the recurrence of three out of four palatal pleomorphic adenoma that underwent malignant transformation occurred after 30 years of the primary tumor.

## Discussion

Due to the rarity of minor salivary gland pleomorphic adenomas, only a limited number of well-documented RPA was found^21^^-^ 33. To fill this gap, this systematic literature review was conducted to investigate the clinical behavior and odds of malignancy of RPA arising in the palate.

The demographic factors of RPA found in this review was in accordance with other studies^7^^,^ 8 ^,^ 34 ^,^ 35: the tumor occurred mainly in women (61.1%), and the average age of patients was 49.3 years old, contrasting only with one study that found higher frequency of RPA in men than women^36^. The patients with RPA were younger at time of diagnosis of the primary pleomorphic adenoma (mean 33 years old – four patients were < 20 years old and six patients were in their twenties), reinforcing the incidence of palatal benign minor salivary gland at earlier age^1^^-^ 3 ^,^ 6 ^,^ 8 ^,^ 36.

Clinically, the RPA on the palate were small, firm, and smooth nodules, asymptomatic, often covered by normal oral mucosa, and measuring up to 3 cm in diameter^21^^,^ 22 ^,^ 28 ^-^ 33, confirming the pattern well-established of the slow growing for this tumor in this location^3^^,^ 37 ^,^ 38. Imaging examinations consistently showed a hypodense maxillary lesion, often with well-defined borders, extending into the maxillary sinus, nasal cavity, and orbital cavity. Additionally, bone involvement was also observed^26^^,^ 27 ^,^ 29 ^,^ 34 ^,^ 36 ^,^ 38. These clinical features align with descriptions found in other studies of primary pleomorphic adenoma in the palate^3^^,^ 37.

The pleomorphic adenoma on the palate is typically treated with surgical resection, which results in low rate of local recurrence^3^^,^ 6. In this systematic review, all RPA were treated by surgical resection^21^^,^ 22 ^,^ 24 ^-^ 33. Of note, the two cases that received adjuvant radiotherapy were treated back in 1986 and underwent malignant transformation. Adjuvant radiotherapy is no longer indicated for benign tumors as it may delay but not prevent recurrence^17^^,^ 34.

Some factors associated with the recurrence of pleomorphic adenoma in different studies, based mainly in parotid gland, were correlated with clinical features of patients and incomplete surgical resection of tumor^7^^,^ 9 ^,^ 14. Previous studies proposed that the higher incidence of primary or recurrent pleomorphic adenoma in women could be associated with higher levels of estrogen^9^^,^ 34. Nevertheless, more studies are required to support the hormonal influence in benign tumor arising from salivary gland. The lack of information about surgical margin in the primary pleomorphic adenoma on the palate was one of the limitations in the present review, not allowing us to analyze the influence of this critical factor on tumor recurrence.

Currently, the time of tumor incubation has gained strength as one of the most determining factors in the recurrence and/or malignant transformation of pleomorphic adenoma^7^^,^ 9 ^,^ 14. This systematic review showed a mean tumor recurrence time of 16.3 years and a mean time of malignant transformation of pleomorphic adenoma of 24.5 years. The recurrence of benign pleomorphic adenoma has been attributed to the presence of microscopic residual disease, primarily resulting from incomplete surgical resection or a contaminated surgical field^3^^,^ 7 ^,^ 9 ^,^ 37. Conversely, the transformation into malignancy is likely driven by inherent tumor factors as cytological and genetic alterations^21^ and this process is time-dependent^14^, confirming the finding of this systematic review. It is important to reinforce that most studies of malignant transformation have focused on samples from pleomorphic adenoma arising from major salivary glands in large medical centers^11^.

Due to the low rate of recurrence and the benign nature of the tumor, long-term patient follow-up is not commonly practiced^7^^,^ 9. Consequently, most studies about RPA were small series and lacked comprehensive information regarding primary lesion (e.g., treatment type, histological features, and surgical margins status)^7^. We found that the average time for tumor recurrence was 16.3 months after primary tumor removal, an average slightly lower than those found by other ones^7^^-^ 9. The odds of detecting a recurrence at 10 and 20 years after primary tumor were 5.57 and 18.78 times higher than at the one-year follow-up time. These results are consistent with other studies suggesting that the length of time the lesion incubates in patients may be associated with recurrence^3^^,^ 7 ^,^ 14. However, in the present literature review, the difficulty in establish whether the pleomorphic adenoma was completely removed and the loss of follow-up of patients after tumor surgery raises the question whether these recurrences are a result of prolonged tumor incubation or the development of primary tumors in the same location.

The RPA on the palate can lead to malignant transformation, negatively impacting the patient’s prognosis^6^^,^ 7 ^,^ 34. In this systematic literature review of 18 cases of RPA, it was found that 22.2% progressed to malignant tumors, a rate considered high compared to other studies^7^^,^ 9 ^,^ 12 ^,^ 38, probably associated with small tumor sample. The recent retrospective study by Choi et al.^14^ comparing malignancy and recurrence of pleomorphic adenoma found no evidence that relapse or repeated surgeries promote malignant transformation of the tumor. Notably, our analysis showed that most of the malignant transformation in the RPA occurred after 30 years of primary tumor removal, in line with other studies indicating a higher risk for malignization in long-standing tumors^13^^,^ 18 ^,^ 27.

The results of this systematic literature review are limited by sample size of RPA in palate, making certain statistical analyses unfeasible and producing possible discrepancies regarding the rate of malignant transformation of this tumor when compared to large minor salivary gland tumor series. Although there is a lack of information in some studies, the data available in this systematic review still suggest a series of coherent arguments. However, it is important to note that further evidence-based research regarding the clinical management of palatal recurrent pleomorphic adenoma is needed to fully evaluate the validity of these arguments.

## Conclusion

The RPA in palate remains a rare event of late occurrence. It affects mainly middle-aged females and carries a risk of malignant transformation. Due to the lack of information, it was not possible to establish whether the recurrence of pleomorphic adenoma in palate was a result of prolonged tumor incubation or if the recurrence were, in fact, new primary tumor in the same location. Although uncommon, the patients with palatal pleomorphic adenoma should be warned about the possibility of recurrence or malignant transformation of tumor at advanced ages.

## Data Availability

Supplementary data are available at: https://zenodo.org/records/10600036 https://doi.org/10.5281/zenodo.10600035
